# Effects of Light, N and Defoliation on Biomass Allocation in *Poa annua*

**DOI:** 10.3390/plants10091783

**Published:** 2021-08-26

**Authors:** Louis John Irving, Sayuki Mori

**Affiliations:** School of Life and Environmental Sciences, University of Tsukuba, Tsukuba 305-8577, Japan; sayukimori@hotmail.com

**Keywords:** light level, N supply, differential growth, shoot:root, root mass fraction

## Abstract

Plants allocate biomass to above- and below-ground organs in response to environmental conditions. While the broad patterns are well-understood, the mechanisms by which plants allocate new growth remain unclear. Modeling approaches to biomass allocation broadly split into functional equilibrium type models and more mechanistically based transport resistance type models. We grew *Poa annua* plants in split root boxes under high and low light levels, high and low N supplies, with N supplied equally or unequally. Our data suggest that light level had the strongest effect on root mass, with N level being more important in controlling shoot mass. Allocation of growth within the root system was compatible with phloem partitioning models. The root mass fraction was affected by both light and N levels, although within light levels the changes were primarily due to changes in shoot growth, with root mass remaining relatively invariant. Under low light conditions, plants exhibited increased specific leaf area, presumably to compensate for low light levels. In a follow-up experiment, we showed that differential root growth could be suppressed by defoliation under low light conditions. Our data were more compatible with transport resistance type models.

## 1. Introduction

In nature, plants must compete for resources, the availability of which varies both temporally and spatially. Shoots compete for light, while roots compete for below-ground resources, such as nutrients and water. The availability of these resources has profound implications for both plant shape and productivity and has been the focus of exhaustive study, yet fundamental questions remain about the mechanisms underlying the regulation of biomass allocation to different organs, and its implications for resource capture.

Given the heterogenous distribution of nutrients in the soil [1], plants have evolved a variety of mechanisms to maximize nutrient capture. Nutrient uptake has been shown to correlate strongly with root length density [2], and root proliferation within nutrient patches is thought to be important both in maximizing the uptake of immobile nutrients [3], and in competitive interactions between plants for even relatively mobile nutrients [4,5]. The C cost of root proliferation has been estimated to be as low as 0.2% of daily photosynthesis [6], although experimental studies have shown that increased root growth within a patch can cause reduced growth outside the patch [3], implying C limitation.

Along with adaptive mechanisms such as differential root growth, root system size has a strong effect on plant–plant competition for resources [7]. Larger root systems are more able to locate valuable nutrient patches in heterogeneous soils, and we would expect plants allocating greater amounts of C to the root system to be more able to compete for scarce resources. Yet, plants must also compete for light, and C allocated to roots is unavailable for the production of above-ground tissues. Poorter and Nagel [8] and Poorter et al. [9] conducted meta-analyses of the factors controlling plant biomass allocation, concluding that light and nutrient supply had opposite effects on root mass fraction (RMF; root mass as a percentage of plant mass); while nutrient supply caused a decrease, RMF tended to increase with light levels. These observations were noted to broadly match the predictions of “functional equilibrium” [10] models of plant growth, such as the “balanced growth hypothesis”, which posit that plants preferentially allocate biomass to organs responsible for harvesting limiting resources [11].

In contrast to empirical functional equilibrium type models, Thornley (1972) proposed the “transport resistance” (TR) model, which offered a more mechanistic model of plant growth based upon the C and N dynamics within the plant [12,13]. This was further developed by Dewar [14], who incorporated greater consideration of nutrient transport systems, although both models highlight the importance of C and N availability in explaining patterns of biomass allocation. Essentially, C or N in excess of immediate requirements may be transported to other plant organs, facilitating growth. A comparison of the two types of models found that mechanistic models were able to explain observed data as well as the functional equilibrium models, but were more able to make specific, testable hypotheses [15]. In a number of papers Andrews and co-workers [16,17,18] used transport resistance-type models to examine the factors influencing plant shoot–root ratio, concluding that shoot-soluble protein concentrations likely have a central role in determining biomass allocation, and speculating that growth is co-limited by local C and N supply. Within this framework, an increase in the N supply would lead to an increase in shoot growth as the N limitation for leaf growth is eased, while increasing light levels would prompt an increase in below-ground biomass allocation as C would become more available for root growth.

Irving et al. [19] investigated the allocation of a ^13^C label to *Poa annua* roots grown in split root boxes, subject to equal and unequal N supply. While root C allocation was responsive to N distribution, the total amount of ^13^C recovered in the roots did not change, despite a 16-fold change in N supply between treatments. It was hypothesized that shoot to root C flux was source-limited, with allocation to individual roots being a separate process. Given that root growth requires C both as a substrate and as an energy source, this lack of difference in shoot-to-root C flux between high and low N supply conditions suggests that soil nutrient conditions have little role in determining total root mass, although the growth of individual roots may be influenced local N availability. The lack of difference in total root mass would seem to contradict functional equilibrium models, which would predict greater resource allocation to those organs taking up limiting resources, in this case N.

To discern the influence of light and N distribution and availability on root growth and biomass allocation we grew *Poa annua* plants under high (HL) and low (LL) light levels, high (HN: 400 µg/2 days) and low (LN: 100 µg/2 days) N levels, with N supplied either equally (HN 200/200; LN 50/50) or unequally (HN 360/40; LN 90/10) and quantified biomass allocation between roots and shoots. *Poa annua* is a small, annual invasive ruderal grass with a worldwide distribution. *Poa annua* has previously been shown to exhibit differential root growth [19], making it suitable for investigating the importance of differential root growth in nutrient uptake from heterogenous environments. We hypothesized that (1) if shoot-to-root C allocation is source limited, light would have a very strong influence on root mass, while the quantity of N supplied and the spatial distribution of that N would have little or no effect; and (2) if shoot-to-root C flux and allocation of assimilation within the root system are independent, we expect to see differential root growth where N is supplied unequally, irrespective of the total N supply or the light environment. In the second experiment, we grew plants under high and low light levels with equal or unequally distributed N, with the plants subject to control conditions, weak or strong defoliation, with the hypothesis that differential root growth may be suppressed under C limited conditions induced by defoliation.

## 2. Results

### 2.1. Experiment One

#### 2.1.1. Shoot Mass

**Figure 1:**
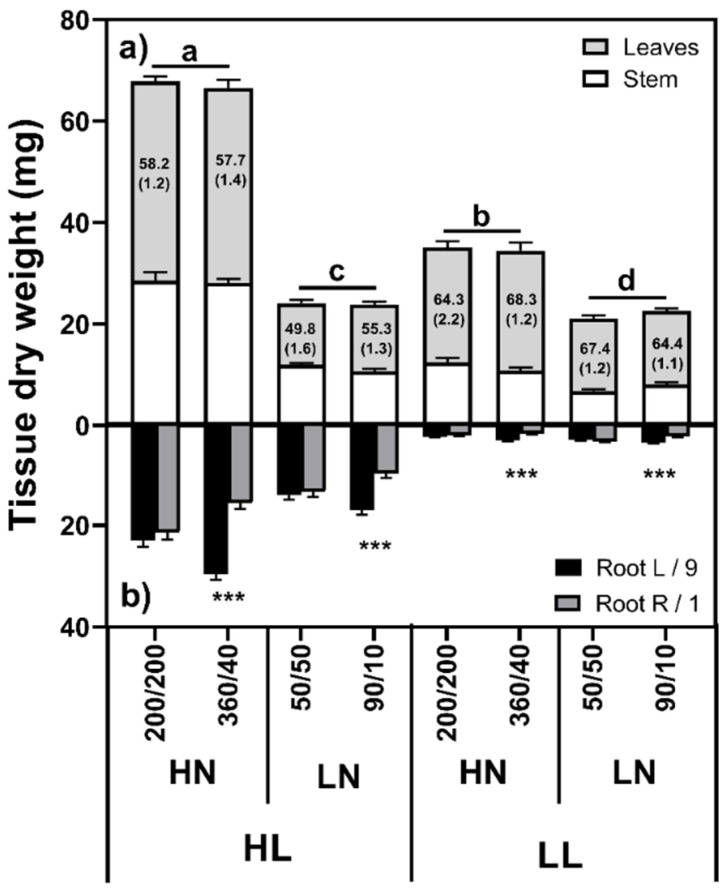
Shoot (**a**) and root (**b**) masses of *Poa annua* plants grown under high (HL) or low light (LL) conditions and supplied either high (HL: 400 µg/2 days) or low (LN: 100 µg N/2 days), distributed either equally (200/200 or 50/50) or unequally (360/40 or 90/10) between root box chambers, where *n* = 12. Numbers in the bars (means ± SE) indicate the percentage of the shoot mass attributable to the lamina. Different letters in the shoot graphs indicate statistically significant differences between light and nitrogen treatments. Asterisks in the root graphs denote significant differences (*p* < 0.001 ***).

Both light level and N supply had significant effects on shoot mass (Figure 1a), with a significant light by N level interaction (F_(1,88)_ = 106.70, *p* < 0.001) suggesting a hierarchical relationship. Increasing the light level under LN conditions led to a modest but significant increase in shoot mass (9.8%; F_(1,88)_ = 5.702, *p* = 0.019), while under HN conditions this increase was much larger (93.2%; F_(1,88)_ = 288.86, *p* < 0.001). Similarly, increasing N levels led to increases under both LL (59.3%, F_(1,88)_ = 135.59, *p* < 0.001) and HL (180.3%, F_(1,88)_ = 689.14, *p* < 0.001) conditions. N distribution had no effect on shoot mass (F_(1,88)_ = 0.028, *p* = 0.867).

A significant light by N level interaction on root mass (Figure 1b; F_(1,88)_ = 34.55, *p* < 0.001) was found, with HL plants having significantly higher root masses than LL plants, both in HN (F_(1,88)_ = 597.74, *p* < 0.001) and LN (F_(1,88)_ = 259.73, *p* < 0.001). N level was also significant, but the differences were smaller (HL: F_(1,88)_ = 33.84, *p* < 0.001; LL: F_(1,88)_ = 6.224, *p* = 0.014). No difference between left- and right-side root masses were found in the equal treatments (*p* > 0.05), while the high N roots had significantly higher masses than the low N roots in all cases (*p* < 0.001). No interaction was found between root mass distribution and either light level (F_(1,88)_ = 1.56, *p* = 0.215) or N level (F_(1,88)_ = 1.09, *p* = 0.299).

#### 2.1.2. N Content/Concentration

**Figure 2:**
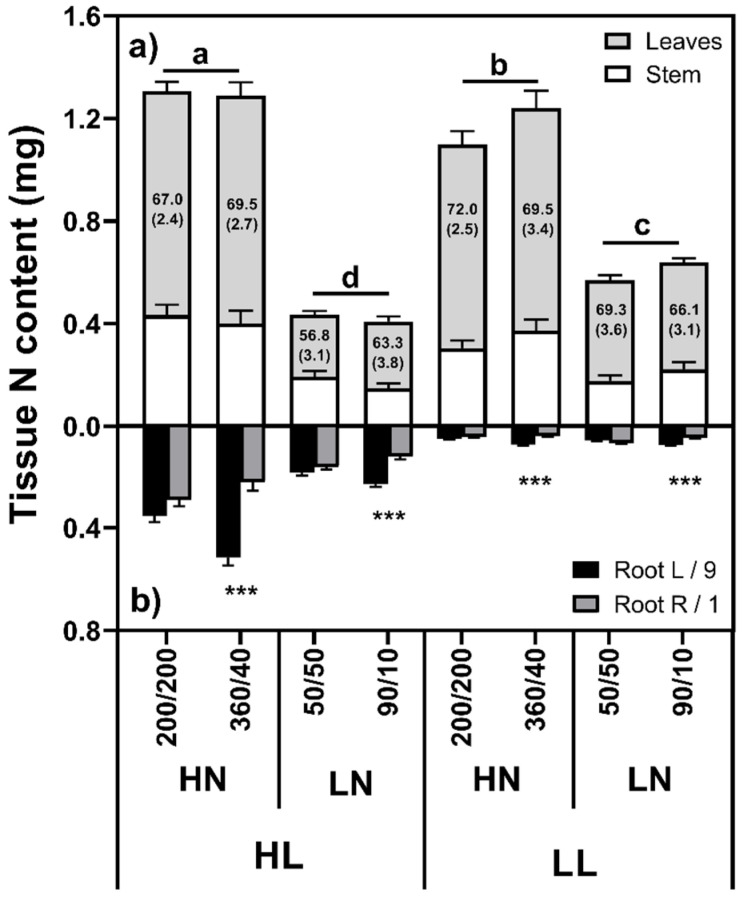
Shoot (**a**) and root (**b**) N contents of *Poa annua* plants grown under high (HL) or low light (LL) conditions and supplied either high (HN: 400 µg/2 days) or low (LN: 100 µg N/2 days), distributed either equally (200/200 or 50/50) or unequally (360/40 or 90/10) between root box chambers, where *n* = 12. Numbers in the bars (means ± SE) indicate the percentage of shoot N content attributable to the lamina. Different letters in the shoot graphs indicate statistically significant differences between light and nitrogen treatments. Asterisks in the root graphs denote significant differences (*p* < 0.001 ***).

Under HN conditions, shoot N content was higher in the HL than LL plants (Figure 2; F_(1,88)_ = 4.24, *p* = 0.042), with the opposite trend noted under LN conditions, such that LL had a higher shoot N content than HL plants (F_(1,88)_ = 50.79, *p* < 0.001). This was not simply due to a change in the distribution between shoots and roots, since the total N content in LL/LN plants was significantly higher than the HL/LN plants (F_(1,88)_ = 83.04, *p* < 0.001). Similarly, the HL/HN plants had a higher total N content than the LL/HN plants (F_(1,88)_ = 252.93, *p* < 0.001), suggesting that not all of the supplied N could be taken up under LL/HN conditions. Root N content was strongly influenced by root mass, although the N concentrations varied among the treatments. Generally, the root N concentration was higher at HL than LL (F_(1,88)_ = 7.61, *p* = 0.007) and under LN conditions (F_(1,88)_ = 133.50, *p* < 0.001). Root N content was not different between left and right roots in the equal treatments (*p* > 0.05) but was significantly greater in the high N than low N roots (*p* < 0.001) in the unequal treatments.

#### 2.1.3. Leaf Area/Specific Leaf Area/Leaf Area Ratio

**Figure 3:**
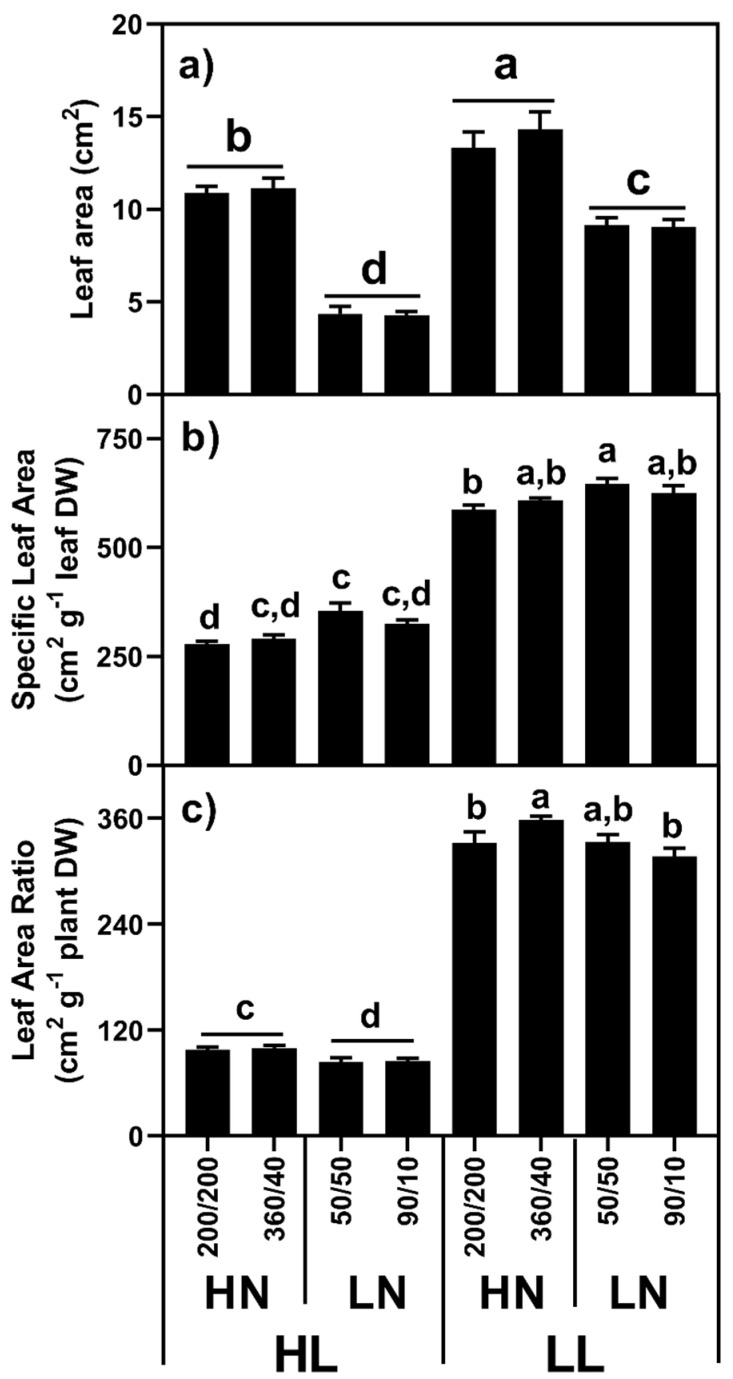
(**a**) Leaf area, (**b**) specific leaf area, and (**c**) plant SLA (leaf area/plant mass) for *Poa annua* plants grown under high (HL) or low light (LL) conditions and supplied high (HN: 400 µg) or low N (LN: 100 µg) equally (200/200 or 50/50) or unequally (360/40 or 90/10) between root box chambers, where *n* = 12. Different letters in the shoot graphs indicate statistically significant differences between light and nitrogen treatments.

Leaf area was strongly influenced by both light and N level, with a strong light by N level interaction (F_(1,88)_ = 44.28, *p* < 0.001; Figure 3). Leaf areas were lower in HL plants in both HN (F_(1,88)_ = 12.35, *p* < 0.001) and LN (F_(1,88)_ = 164.30, *p* < 0.001) conditions, and higher in HN plants at both HL (F_(1,88)_ = 257.20, *p* < 0.001) and LL conditions (F_(1,88)_ = 45.35, *p* < 0.001).

Light level had a very strong effect on specific leaf area (F_(1,88)_ = 1312.03, *p* < 0.001), with LL plants exhibiting values approximately double those of HL plants (615.5 vs. 312.0 cm^2^ g^−1^ DW). HN plants tended to have slightly lower SLA values than LN plants both when supplied N equally (F_(1,88)_ = 32.70, *p* < 0.001) and unequally (F_(1,88)_ = 4.77, *p* = 0.032). Under LN conditions, plants supplied N equally had slightly higher SLA values than those supplied N unequally (5.2%; F_(1,88)_ = 4.47, *p* = 0.037).

We calculated the leaf area ratio (LAR) as a measure of the balance between C fixation and demand, assuming that leaves represent the primary organ for photosynthetic C gain. Similar to SLA, light level had a very strong effect on LAR, both under HN (F_(1,88)_ = 1296.18, *p* < 0.001) and LN (F_(1,88)_ = 1517.30, *p* < 0.001), while N level had a small effect under HL conditions (F_(1,88)_ = 21.53, *p* < 0.001). Under HN conditions, the LL plants had a LAR around 3.5 times larger than the HL plants, while under LN conditions, LL plants were 3.85 times larger. Given the differences in light levels, this suggests an approximately equal photon capture per unit biomass between the HL and LL plants.

#### 2.1.4. Root Mass Fraction

Root mass fraction was strongly affected by both light and N supply, although N distribution did not have an influence. High light levels caused an increase in RMF (F_(1,88)_ = 987.45, *p* < 0.001), while high N caused a significant decrease (F_(1,88)_ = 143.87, *p* < 0.001).

**Figure 4:**
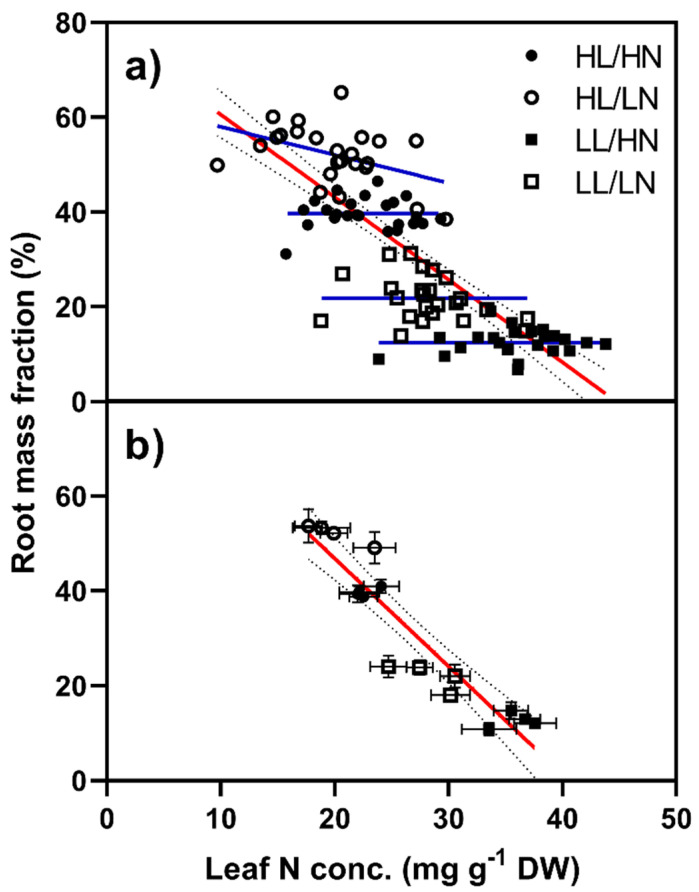
Root mass fraction plotted against leaf N concentration for *Poa annua* plants grown under high (HL) or low light (LL) conditions and supplied high (400 µg) or low N (100 µg). The red line represents a regression of RMF against leaf N concentration. In (**a**) each datapoint represents an individual plant and the blue lines represent regression lines for each treatment while in (**b**) each datapoint represents the mean of six replicates, with the error bars representing standard errors.

Taken together, a significant negative relationship (F_(1,94)_ = 165.9, *p* < 0.001, r^2^ = 0.638) was found between leaf N concentration and RMF (Figure 4a). When the data were split based on light and N levels, no relation between leaf N concentration and RMF was found in three of the four groups, with the HL/LN plants having the only significant relationship (F_(1,22)_ = 5.33, *p* = 0.031). Previous studies tended to use means of several replicates, rather than raw data. To check whether this had an effect, we split each treatment group into four sets of six replicates and calculated the means and SE for both RMF and leaf N concentration. This resulted in a strong negative relationship between leaf protein concentration and RMF (Figure 4b; F_(1,14)_ = 97.88, *p* < 0.001, r^2^ = 0.875).

#### 2.1.5. Experiment Two

Defoliation caused a decrease in plant mass under both light intensities (Table 1), with a light by defoliation interaction (F_(2,130)_ = 88.59, *p* < 0.001). While defoliation led to a decrease in shoot mass at both light levels, under HL conditions, defoliation intensity had no effect on shoot mass, while under LL conditions the shoot mass was lowest in plants subject to the strongest defoliation treatment. Broadly speaking, the shoot masses of control HL and LL plants were similar to the HN plants in experiment one.

#### 2.1.6. Root Mass

Both light level and defoliation had strong effects on root mass, although a light by defoliation interaction (F_(2,130)_ = 3.221, *p* = 0.043) was noted. Under HL conditions, root masses were a function of defoliation frequency, with the lowest root mass noted under strong defoliation. However, under LL conditions, while defoliation led to a decrease in root mass, no difference was evident between plants experiencing weak or strong defoliation (*p* = 0.496).

No difference in root mass between chambers was noted in the plants receiving N equally to the two chambers. Under HL conditions when N was supplied unequally, root masses were distributed in a 60:40 manner between the HN and LN chambers. Under LL conditions a difference in root mass between HN and LN chambers was only found in control plants; no difference in root mass between chambers could be found under defoliation.

Under HL conditions, RMF decreased with increasing defoliation intensity (F_(2,130)_ = 18.20, *p* < 0.001), while under LL, defoliation caused an increase in RMF (F_(2,130)_ = 12.73, *p* < 0.001) presumably due to the decrease in shoot mass. No difference in RMF was found between strong and weak defoliation treatments under LL (*p* = 0.848).

## 3. Discussion

### 3.1. Root Growth Is Mainly Controlled by Light Level

Based on Irving et al. [19] we hypothesized that shoot-to-root C flux, and therefore root growth, would be source-limited by C fixation rate, while N supply and distribution would have no effect. Our results largely confirmed the strong influence of light level, with high light (HL) plants producing greater root masses than their low light (LL) counterparts. Contrary to expectations, a significant light by N level interaction on root mass was found, with higher root masses in HN plants under HL, and LN plants under LL conditions. Comparing F-values, differences arising from light level were over an order of magnitude stronger than the N treatments, and these N effects were presumed to be a result of increased plant mass under HL conditions, and a shift in C allocation towards the shoot under LL conditions. However, it is plausible that the reduction in root biomass under LL conditions was due to the respiratory costs of N uptake and assimilation [20]. Generally, the root mass fraction was approximately 10% higher in LN plants at both light levels, largely as a result of decreased shoot growth. Our results fit well with Thornley’s Transport Resistance model of plant growth [12], and are consistent with our understanding of photoassimilate transport [21,22], with presumably higher C export rates from the shoot giving rise to higher root masses under high light. Given the relatively modest influence of N on root mass, our results fit less well with Functional Equilibrium type models, which would predict increased root growth under N limited conditions.

### 3.2. C Allocation within the Root System

Once C is transported to the roots, for differential growth to occur it must be allocated to those roots encountering high nutrient conditions. While local root growth was generally responsive to differential N supply, the total root mass did not differ between plants supplied N equally or unequally. This suggests that while the total C supply to the roots is controlled by above-ground factors, C allocation within the root system is likely controlled by source–sink interactions resulting from local conditions [3]. Studies of source–sink interactions have shown differential phloem unloading rates to be a key driver of C partitioning between sinks [21,23]. C unloading into root cells is thought to be mediated by the size of the soluble carbohydrate pool [24], and it seems likely that N supply may cause an increase in respiration rates, due to the energy costs of uptake and assimilation [25]. Previous studies have shown that a half or more of C used in new growth was fixed within the previous 24 h [26], suggesting a tight coupling of C fixation and growth. Modelling of the C balance of *Lolium perenne* roots similarly suggests a close link between photoassimilate supply, and the growth rate of young, actively growing roots [27].

Our LL treatment imposed a severe C deficiency on plants. While the plants could seemingly compensate for low light over the experiment by increasing SLA and leaf area [9], when the accumulation of leaf area was suppressed by defoliation, plants were unable to engage in differential growth—presumably due to intense C limitation, where the roots could either not grow, or grew only minimally. Differential root growth occurred wherever the plants were able to grow, suggesting that differential root growth may be inherent feature of growth in *Poa annua*. Our results are consistent with previous defoliation studies showing strong declines in shoot to root C flux, as photoassimilate is reassigned to support leaf regrowth [28]. Ecologically, we may expect that both defoliation and irradiance level may have important implications for plant–plant competition in heterogeneous environments.

### 3.3. N Uptake and Shoot Growth

Shoot N content was primarily controlled by N supply and was broadly similar between light levels within each N treatment. Root N content, conversely, was mainly influenced by light levels since tissue N concentrations did not differ greatly between HN and LN conditions. Despite their low root masses, our LL plants were able to accumulate similar levels of N with HL plants. While previous studies have shown that N uptake is strongly influenced by root length [2], and some have attributed growth declines to insufficient uptake capacity from N patches [19,29,30,31], other studies have suggested that where plant growth rates are limited by some other factor, N uptake is purely a function of supply [32]. Mechanistically, these high N uptake rates seen in the LL/HN plants may result from a stimulation of local inflow [6,33], or an increase in specific root length [34], such that each unit mass of roots is exploring a greater soil volume.

Compared with the LL/LN plants, increasing N availability had a larger positive effect on shoot growth than increasing light levels. We interpret this as N level being more limiting for shoot growth under LN conditions, and light more limiting under HN conditions. The effects of both light and N on plant growth are unsurprising, given their importance in photosynthesis [35,36]. Light similarly influenced the composition of the shoot materials, with LL plants partitioning a greater fraction of their shoot mass to leaf lamina (65–70%) compared with HL plants (50–60%). N had no discernible influence on the share of shoot growth between lamina and pseudostem tissues.

Leaf area was influenced by both light and N supply, with higher leaf areas under LL and HN conditions. These high leaf areas were achieved in the LL plants by a significant increase in SLA, with N level having only a small influence on SLA, patterns consistent with previous reports [9]. We also calculated the leaf area ratio (LAR) to help us evaluate the relationship between C fixation and demand, assuming that the former is a function of leaf area, while the latter a function of plant mass. Broadly speaking, LL plants had approximately four-times more leaf area supporting each unit of plant tissue, presumably to compensate for the low photon flux density.

### 3.4. Shoot to Root Ratio

Historically, measures such as shoot:root (S:R) have been used to describe biomass allocation between plant parts, with these divisions broadly reflecting the balance between light and nutrient harvesting components. Poorter and Nagel [8] proposed that dividing the plant into leaves, shoots, and roots was preferable due to their different roles in the plant, and that the allocation of biomass to these tissues should be expressed as fractions of the whole plant. Irrespective of which of these two metrics we use, the importance of nutrient supply and light in determining biomass allocation is inescapable [9,18], with nutrient supply causing a shift in biomass allocation towards the shoots, while increasing light levels causes an increase in allocation to the roots.

Changes in root mass fraction are frequently attributed functional importance. For example, increases in RMF under nutrient limitation are portrayed as the plant investing in nutrient foraging capacity. In our plants, increases in RMF resulting from low nutrient supply were almost entirely attributable to declines in shoot mass, with the absolute root masses being relatively unchanged within light levels. Similar patterns can be seen in historical studies, with Brouwer [10] noting that increasing N supply caused increased shoot growth while having little effect on root masses, except under low light conditions. Similarly, Vessey and Layzell [20] found that while root growth rate decreased slightly with increasing N supply in soybean, this was likely due to the increasing respiratory costs of N assimilation, as the C flux to the roots did not differ between treatments. Our example of high RMF under N-deficiency can be framed in different ways; as smaller plants investing a greater proportion of their biomass into the roots, or, if we consider root and shoot growth to be somewhat independent, as a reduction in shoot growth while the root mass remains relatively constant. In the second framing, increases in RMF would be considered as more of a result of mathematical necessity, rather than indicating some change in root behavior.

At a macro level, our root mass allocation vs. shoot N concentration data mirrors previous studies (e.g., [18]), with a negative relationship between leaf N concentration and RMF (i.e., an increasing S:R). However, this relationship broke down when considering each treatment group individually, with no relationship between leaf N concentration and RMF in three of the four groups (Figure 3a). The HL/HN group did exhibit a negative relationship between leaf N concentration and RMF, but the gradient was significantly different to the macro-level regression. Within groups, the highest measured leaf N concentrations were often around twice the lowest values, suggesting there should have been sufficient variability within the groups for these relationships to manifest, assuming a causal relationship between leaf protein concentration and S:R [16]. We also noted a high degree of overlap between the datasets, such that for a leaf N concentration of 25 mg N g^−1^ DW, the RMF varied from around 10% in the LL/HN plants, to nearly 60% in the HL/LN plants. It should be noted that previous studies have correlated root mass fraction against soluble protein concentration, while we correlated total leaf N, which includes insoluble N, which is typically not metabolically active. This said, chloroplast N can account for up to 80% of leaf total N in pea plants [37], and it seems not unreasonable to expect the proposed correlations to manifest, whichever measurement of leaf N we use. Previous studies have tended to plot out means, rather than raw data [16,18]. Where we plotted out the means for six replicates (Figure 4b), the strength of the relationship (r-squared value) increased substantially (0.875 vs. 0.638) compared to the raw data. Although the use of means greatly clarifies the relationship between leaf N levels and RMF, it also has the effect of obscuring the true variability of the underlying data, and there is a risk it may give readers a false impression of the strength of the relationship.

Thus, given the variability of the raw data, and the lack of relationship between leaf N levels and RMF within groups, RMF is more reasonably explained in our experiment by the individual influences of light on root mass and N supply and light level on shoot growth. This said, Andrews et al. [18] covered a wide range of species and nutrient conditions in their analysis of the literature, noting species specificity in some trends, including differences between ammonium vs nitrate nutrition, and it is plausible that our results represent only some part of this diversity of response.

We started this section with a discussion of functional explanations for changes in RMF, finding functional equilibrium models focusing on nutrient uptake to be unconvincing. An alternate functional explanation for comparatively high root masses under low nutrient conditions would be to consider their role as C sinks. As sessile organisms, plants have no ability to avoid unfavorable conditions, including excess light. Under high light conditions, plants can experience feedback repression of photosynthesis due to carbohydrate build up, leading to light stress and potentially photodamage [38]. Export of excess C to roots represents a potential strategy of avoiding these issues. As well as the C costs of root growth, maintenance respiration represents a second, ongoing C sink [27]. Where a plant has insufficient nutrients to support the production of new leaves, root growth may be an excellent way for the plant to balance its C budget.

## 4. Materials and Methods

*Poa annua* seeds were germinated on moist tissue for one week before being transplanted to sand-filled seedling trays. The young plants were provided half-strength Long Ashton solution and grown with a 14/10 (L/D) photoperiod under medium light levels (approx. 300 µmol photons m^−2^ s^−1^, supplied by commercially available full-spectrum LED growth lights (KingLED, Shenzhen, China)) for 18 days in experiment one and 22 days in experiment two. At this point, the plants were transplanted into split root boxes as described previously in Irving et al. [19]. The plants were grown under the same light conditions and provided 5mL of N-free Long Ashton solution to each chamber of the root box every two days and grown for a further six days to allow plant establishment and the depletion of any internal N stores before the start of treatments.

### 4.1. Experiment One

This experiment aimed to test the hypothesis that light level would have a strong influence on the total mass of roots produced by a plant, while both N supply and distribution would have little or no influence on root biomass production. The experiment had a 2 [light] × 2 [N supply] × 2 [N distributions] design with 12 replicates per treatment.

After the six-day acclimation period following transplanting to the root boxes, half the plants were grown under high light conditions (HL; 500 µmol photons m^−2^ s^−1^), and the other half under low light (LL; 100 µmol photons m^−2^ s^−1^) with a 14/10 (L/D) photoperiod. Within each light level, half the plants were supplied high N (HN; 400 µg/2 days), and half supplied low N (LN; 100 µg N/2 days) as ammonium sulfate supplemented to 10 mL (5 mL to each root chamber) of an N-free Long Ashton nutrient solution. The N distributions used were 50/50 (denoted 200/200 for HN, and 50/50 for LN), and 90/10 (denoted 360/40 and 90/10).

After 18 days of treatment, the plants were destructively harvested. Plants were split into leaves, pseudostem, and two sets of roots. The leaves were scanned using a flatbed scanner (Canon Canoscan LiDE 220) and the leaf areas were measured using ImageJ [39]. The shoot and washed roots were oven-dried for a minimum of 96 h before being weighed. Shoot and root N concentrations were quantified using a micro-Kjeldahl digest method, with the N concentration determined using Nessler reagent [40].

### 4.2. Experiment Two

This experiment aimed to test the hypothesis that differential root growth could be suppressed in LL plants, if they were prevented from compensating for the reduction in light levels. The experiment had a 2 [light] × 2 [N distribution] × 3 [defoliation levels] fully factorial design, with 12 replicates per treatment.

As in experiment one, plants were grown under high light (HL; 500 µmol photons m^−2^ s^−1^) or low light conditions (LL; 100 µmol photons m^−2^ s^−1^). Only a single N level was used (400 µg/2 days), with the N supplied in either a 50/50 or 90/10 distribution between the two root chambers. After the six-day acclimation period following transplanting to the root boxes, the N distribution and defoliation treatments were commenced. One group of plants at each light level was designated as controls and was not subject to defoliation. The other two groups were designated as “weak” and “strong” defoliation and were trimmed to 50 mm from soil level using scissors. The strong defoliation treatment was defoliated every two days, while the weak defoliation treatment was defoliated on day 0, 4, then every subsequent 6 days.

The plants were destructively harvested on day 24, at the end of a regrowth cycle for the weak defoliation plants. The plants were separated into shoots and two sets of roots, before being dried and weighed as in experiment one.

### 4.3. Data Analysis

Differences between shoot data were analyzed using the Univariate GLM command, while paired root data were analyzed using the repeated-measure GLM in IBM SPSS Statistics for Windows (version 27). Data were square root or log-transformed on an as-necessary basis to ensure homoskedasticity and data normality. Error bars for paired samples were corrected according to the method of Loftus and Masson [41]. Regression analysis was performed in Graphpad PRISM (v9) software.

## 5. Conclusions

In conclusion, our data suggest that root mass in *Poa annua* is largely controlled by light level, while shoot mass was a function of both N availability and light, with N being more important. The allocation of growth within the root system was strongly influenced by N distribution. Our data were consistent with the model of Thornley [12,13], but not functional equilibrium models, which would have predicted increased root growth in response to low nutrient supply. Root mass fraction tended to be higher in low N plants, but this was mainly attributable to decreased shoot mass, with root masses being largely unaffected by N supply. As in previous studies, a negative relationship was found between root mass fraction and leaf N concentration, although this was not found within each of the individual treatments. It was concluded that the separate effects of N and light intensity were sufficient to explain this phenomenon.

## Figures and Tables

**Table 1:** Effect of strong (every 2 days) or weak (every 6 days) defoliation on the shoot and root mass (mg) in *Poa annua* plants grown under high and low light, where *n* = 12. In the plants supplied N equally, the roots are denoted as L and R, while in the plants supplied N unequally, they are denoted 9 and 1. The L:R ratio shows the distribution of roots between the two root box chambers. For shoot mass, different letters indicate statistically significant differences between groups. *p*-values represent paired comparisons between root masses in each split root box chamber. Bold letters represent statistically significant differences at *p* ≤ 0.05.

Light	Defoliation	N Distribution	Shoot	L/9	R/1	*p*-Value	L:R Ratio	RMF (%)
High	None	Equal	68.6 (2.4) ^a^	23.4	22.2 (1.0)	0.651	52:48 (2)	39.5 (1.4) ^a^
		Unequal	72.4 (3.5) ^a^	26.7	17.2 (0.7)	**0.006**	61:39 (2)	37.7 (1.0) ^a^
	Weak	Equal	49.6 (1.5) ^b^	13.9	14.9 (0.9)	0.224	47:53 (3)	36.4 (1.2) ^a^
		Unequal	52.3 (1.7) ^b^	14.1	9.6 (0.8)	**<0.001**	60:40 (4)	30.9 (1.4) ^b^
	Strong	Equal	47.7 (1.6) ^b^	10.3	11.6 (0.6)	0.507	46:54 (3)	30.7 (1.7) ^b^
		Unequal	49.2 (1.0) ^b^	13.7	7.7 (0.5)	**0.005**	65:35 (3)	29.8 (1.4) ^b^
Low	None	Equal	28.6 (1.7) ^c^	2.0	2.1 (0.2)	0.616	48:52 (6)	12.2 (0.7) ^c^
		Unequal	29.6 (2.1) ^c^	2.3	1.4 (0.2)	**0.001**	62:38 (4)	11.0 (0.5) ^c^
	Weak	Equal	8.7 (0.3) ^d^	0.9	0.7 (0.1)	0.824	54:46 (5)	15.1 (1.3) ^c^
		Unequal	8.1 (0.3) ^d^	0.8	0.7 (0.1)	0.356	56:44 (5)	15.4 (1.2) ^c^
	Strong	Equal	7.4 (0.4) ^e^	0.7	0.7 (0.1)	0.227	49:51 (3)	15.9 (0.8) ^c^
		Unequal	6.9 (0.4) ^e^	0.7	0.6 (<0.1)	0.083	54:46 (2)	15.7 (0.7) ^c^

## Data Availability

These data are available at http://doi.org/10.5281/zenodo.5131522.
